# The Interaction between Enterobacteriaceae and Calcium Oxalate Deposits

**DOI:** 10.1371/journal.pone.0139575

**Published:** 2015-10-08

**Authors:** Evan Barr-Beare, Vijay Saxena, Evann E. Hilt, Krystal Thomas-White, Megan Schober, Birong Li, Brian Becknell, David S. Hains, Alan J. Wolfe, Andrew L. Schwaderer

**Affiliations:** 1 The Research Institute at Nationwide Children’s Hospital, Center for Clinical and Translational Research, Columbus, Ohio, United States of America; 2 Loyola University Chicago, Stritch School of Medicine, Department of Microbiology and Immunology, Chicago, Illinois, United States of America; 3 Nationwide Children’s Hospital, Division of Urology, Columbus, Ohio, United States of America; 4 Nationwide Children’s Hospital, Division of Nephrology, Columbus, Ohio, United States of America; 5 Lebonheur Children’s Hospital, Division of Nephrology, Memphis, Tennessee, United States of America; Emory University, UNITED STATES

## Abstract

**Background:**

The role of calcium oxalate crystals and deposits in UTI pathogenesis has not been established. The objectives of this study were to identify bacteria present in pediatric urolithiasis and, using *in vitro* and *in vivo* models, to determine the relevance of calcium oxalate deposits during experimental pyelonephritis.

**Methods:**

Pediatric kidney stones and urine were collected and both cultured and sequenced for bacteria. Bacterial adhesion to calcium oxalate was compared. Murine kidney calcium oxalate deposits were induced by intraperitoneal glyoxalate injection and kidneys were transurethrally inoculated with uropathogenic *Escherichia coli* to induce pyelonephritis

**Results:**

*E*. *coli* of the family Enterobacteriaceae was identified in patients by calcium oxalate stone culture. Additionally Enterobacteriaceae DNA was sequenced from multiple calcium oxalate kidney stones. *E*. *coli* selectively aggregated on and around calcium oxalate monohydrate crystals. Mice inoculated with glyoxalate and uropathogenic *E*. *coli* had higher bacterial burdens, increased kidney calcium oxalate deposits and an increased kidney innate immune response compared to mice with only calcium oxalate deposits or only pyelonephritis.

**Conclusions:**

In a murine model, the presence of calcium oxalate deposits increases pyelonephritis risk, likely due to preferential aggregation of bacteria on and around calcium oxalate crystals. When both calcium oxalate deposits and uropathogenic bacteria were present, calcium oxalate deposit number increased along with renal gene transcription of inner stone core matrix proteins increased. Therefore renal calcium oxalate deposits may be a modifiable risk factor for infections of the kidney and urinary tract. Furthermore, bacteria may be present in calcium oxalate deposits and potentially contribute to calcium oxalate renal disease.

## Introduction

Approximately 10% of people develop a kidney stone during their lifetime[1]. Calcium oxalate (CaOx) accounts for 74% of stones[2]. Considerable overlap in CaOx urine super-saturation exists between individuals with and without kidney stones; therefore, urine chemistries cannot be the only factor in stone formation[3]. Magnesium-ammonium-phosphate (struvite) stones form as a result of a urinary tract infection (UTI) with a urease-producing bacterium and are a conglomeration of bacteria, struvite crystals and protein matrix[4]. Furthermore emerging evidence indicates an interaction between bacteria and CaOx kidney stones. First, patients with kidney stones are more likely to have UTIs than the general population[5, 6]. Second, in previous studies, bacteria have been cultured from 19–32% of CaOx stones, with non-urease-producing *E*. *coli* most commonly present[7, 8]. *E*. *coli*, a major member of the Gram-negative bacterial family Enterobacteriaceae, is a contributor to a wide range of kidney pathologies ranging from pyelonephritis to kidney allograft rejection[9, 10]. Despite its role in other pathologies, the association between *E*. *coli* and CaOx disease has not been extensively investigated.

Traditionally, the urinary tract has been considered sterile; however, high throughput sequencing technology and enhanced quantitative urine culture (EQUC) protocols have identified a urinary microbiome in culture-negative urine samples [11–13]. Whether kidney stones also have a microbiome has not yet been evaluated. The objectives of this study were to determine whether bacteria are present in human CaOx kidney stones and, using *in vitro* and *in vivo* models, evaluate potential associations between *E*. *coli* and renal CaOx deposits.

## Materials and Methods

### Human studies

#### Patients and sample collection

Human studies were approved by the Nationwide Children’s Hospital (NCH, Columbus, OH) Institutional Review Board (IRB13-00709) and adhered to the *Declaration of Helsinki*. Inclusion criteria consisted of patients with a kidney stone removal procedure by the NCH Urology Division. Exclusion criteria consisted of a history of known struvite, cysteine or uric acid stones. Written consent was obtained from legal guardians if the research subject was a minor additionally assent was obtained if children were ≥ 9 years of age. Following kidney stone removal, the urologist bisected the stone. Half of the stone was placed in a sterile eppendorf tube and the other half was sent for routine clinical stone analysis (Louis C. Herring and Company, Orlando FL) Upper tract and bladder urine was collected with the kidney stones during the stone removal procedure. Immediately following sample acquisition, study staff was notified to process the samples. Patient history including urine chemistries were recorded and urine oxalate, calcium and citrate to creatinine ratios were compared to reported normal ranges[14, 15].

#### Sample processing

Stone fragments were rinsed with sterile PBS, and then homogenized in a bullet blender (Next Advance, Averill Park, NY). Stone homogenate and urine were aliquoted between urine C&S preservative (Becton Dickenson, Franklin Lakes NJ, United States) and 10% AssayAssure universal urine collection media (Thermo Scientific, Waltham MA) for culture and sequencing analysis, respectively. Sterile PBS with and without bullet blender beads was used as control to evaluate for contamination during stone processing.

#### Enhanced Quantitative Urine Culture (EQUC)

0.1 mL of urine or kidney stone homogenate was inoculated onto BAP, Chocolate, Colistin, Naladixic Acid (CNA) agars or CDC anaerobe 5% sheep blood agar plates (BD BBL™ Prepared Plated Media) and EQUC was performed as previously described[12]. The detection level was 10 CFU/mL. Matrix Assisted Laser Desorption Ionization Time of Flight Mass Spectrophotometry (MALDI-TOF MS) was used to identify the bacterial isolates.

#### DNA isolation and 16S amplicon sequence analysis

DNA extraction along with 16S rRNA gene library generation and DNA sequencing with a MiSeq desktop sequencer (Illumina, San Diego, CA) were performed as described previously [11]. The DNA from the kidney stones was extracted as described previously with the exception that we used lysis buffer ALT in the Qiagen DNeasy kit. All samples were processed in duplicate, replicas were comparable; therefore, only the first replica was used for downstream analysis. Since urine is a low biomass environment, genera were reported only if a group representing 10% of the total reads in at least one sample; the rest of the reads are reported as “other” [11, 12].

### *In vitro* studies

CaOx monohydrate and dihydrate crystals were generated as previously described[16]. For the *in vitro* binding assay 100μg of CaOx or control silicon dioxide crystals (Strem Chemicals, Newburyport, MA) were mixed with 1x10^5^ colony forming units (CFU)/ml GFP-labeled uropathogenic *E*. *coli* (UPEC, a gift from Matthew Mulvey, University of Utah) and incubated in Luria Broth (LB) in a 37°C orbital shaker (Thermo Scientific, Waltham MA). Silicon dioxide crystals were chosen as a control because they are commercially available and similar in size (10–20 nm) to the generated CaOx crystals. Additionally, silicon has been reported to cause kidney stones in human and is a well-established kidney stone component in veterinary medicine [17, 18].

To quantify the area of crystals, Keyence BZ-II analyzer software was used. The area of the crystals was outlined using the Freehand Line feature of the Area Measure function. Within the area of the crystal, GFP-expressing *UPEC* were then counted within the area of the stone using the Count function. The same technique was employed for the background, outlining an area of the background using the Freehand Line and then counting the *E*. *coli* within the outlined area. A ratio of *E*. *coli* per μm^2^ area was then generated by dividing the number of *E*. *coli* by the corresponding outlined area

### Murine studies

#### Mice

*In vivo* murine studies were approved by the Institutional Animal Care and Use Committee (IACUC) protocol AR12-00067 and adhered to NIH Guide for the Care and Use of Laboratory Animals or the equivalent. C57BL/6 mice (Jackson lab, Bar Harbor, Maine, Stock no. 000664) aged 6–10 weeks of were used for induction of pyelonephritis and/or CaOx nephropathy. Mice were euthanized by CO_2_ inhalation at the end of the study for assessment of kidney bacterial burden, CaOx deposits and kidney innate immune gene transcript evaluation.

#### Induction of kidney CaOx induced nephropathy and pyelonephritis

Kidney CaOx deposits were induced in mice by injecting sodium glyoxalate (80mg/kg/day of glyoxalate (Sigma Aldrich, St Louis, MO) intraperitoneally (i.p.); this causes CaOx deposits to appear by 3 days and to peak at day 6 [19]. Equivalent normal saline was used as negative control. Experimental pyelonephritis was induced by inoculating 10^8^ colony forming units (CFU) of the uropathogenic *E*. *Coli* (UPEC strain; CFTO73) transurethrally with a second inoculum of the same size performed 4 hours later[20].

### Experimental groups; innate immune response induced by CaOx deposits

1. CaOx deposits: Sodium glyoxalate injection i.p. for 6 days.
2. Negative control: Normal Saline (NaCl) injections i.p. for 6 days.

Male C57BL/6 mice were used for this experiment and evaluated at peak kidney CaOx deposition. The goal was to identify candidate innate immune genes to study the interaction of CaOx deposition and UPEC at an early time point.

### Experimental groups; early innate immune response with CaOx deposits and UPEC, alone or in combination

**Fig 1:**
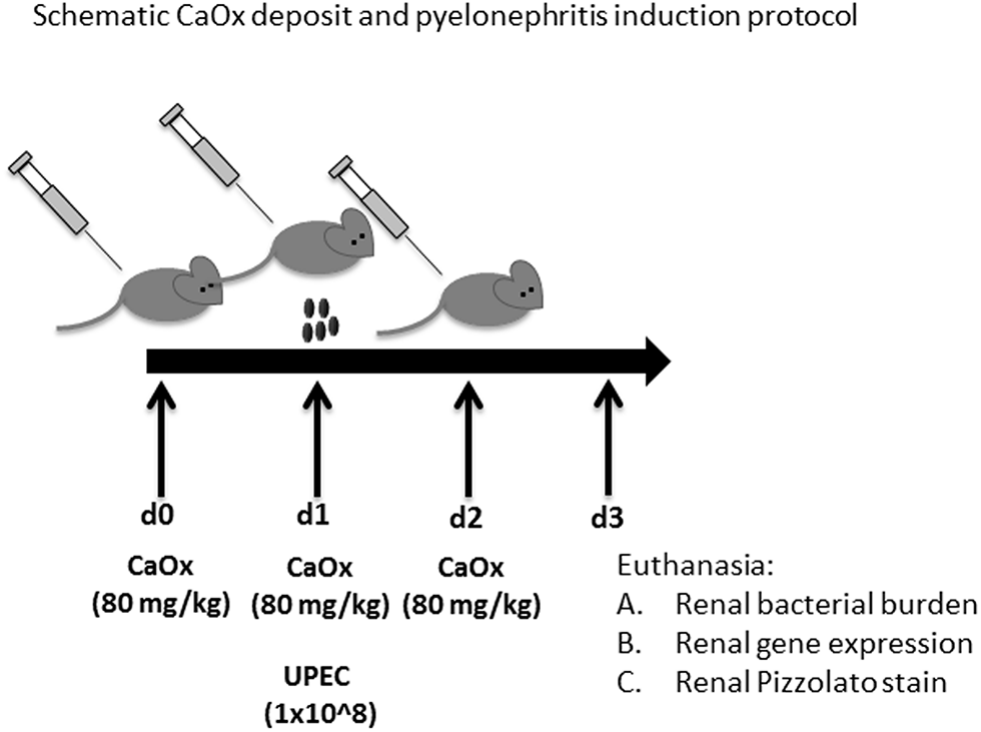
Induction of CaOx deposits and experimental pyelonephritis in mice.

1. Kidney CaOx deposits: Sodium glyoxalate injection i.p for 3 days.
2. UPEC inoculation: NaCl injections i.p. for 3 days, UPEC inoculation on day 1.
3. Kidney CaOx deposits and UPEC inoculation: Sodium glyoxalate injection i.p. for 3 days and 10^8^ CFU UPEC inoculation on day 1. A schematic of the experimental time course for this group is presented in Fig 1.
4. Negative control: NaCl injections i.p. for 3 days.

The 3-day CaOx treatment/2-day experimental pyelonephritis time period was chosen because C57BL/6 mice clear infections by 3rd day [20]. Female mice were used for this experiment because UPEC inoculation is not possible with the male murine anatomy.

#### Outcome studies

Kidneys were bisected longitudinally and bacterial burden was determined by plating serial dilutions of half-kidney homogenate on a LB plate. The other half kidney was used for CaOx deposit and/or pyelonephritis confirmation or snap frozen for RNA extraction. Kidney deposits were visualized and quantified with Pizzolato staining using BZII analyzer software (S1 Fig). Kidneys were cultured for bacterial burden as previously described[21]. The local renal CaOx induced innate immune response was determined using the RT^2^ Profiler Antibacterial Response Array (Qiagen, Valencia, CA, Catalog no. PAM148Z) according to the manufacturer’s directions. Gene expression was normalized using a panel of five housekeeping genes (*Actb*, *B2m*, *Gapdh*, *Gusb* and *Hsp90ab1*). Targeted PCR was performed using KicqStart SYBR green primers (Sigma-Aldrich, St Louis, MO) for candidate innate immune genes identified in RT^2^ profiler assay. *Gapdh* (Real Time Primers, LLC, Elkins Park, PA) was used as a housekeeping gene for targeted PCR. All PCR reactions were performed on a 7500 PCR system (Applied Biosystems, Carlsbad, CA).

#### Statistics

Statistical analysis was performed and graphs were generated using Prism software (GraphPad Software Inc, La Jolla, CA). The D’Agostino-Pearson Omnibus Test was used to determine if data was normally distributed. Differences between groups were compared with the Student’s T-test or One-Way Anova if data was normally distributed; otherwise, the Mann Whitney or Kruskal Wallis methods were used. Percentages were transformed to arcsine values for analysis. Arrays were analyzed with the RT^2^ Profiler Data Analysis Software. For targeted PCR, expression was quantified using the 2^-ΔΔCt^ method and normalized values to the reference gene *Gapdh*.

## Results

### Patients

Five patients were enrolled (Table 1) between August 2013 and February 2014. Patient 5 did not have documented CaOx stones, but had a history of hypercalciuria and a urine pH of 6, inconsistent with struvite. Only patients 1 and 2 had a history of UTIs. No patients were diagnosed with a UTI during or 30 days prior to the stone removal procedure. However, patient 1 was treated for a UTI following stone removal.

**Table 1:** Patient Characteristics.

Subject	Age (yrs) /sex	Dx	Procedure	Stone analysis	24 hr urine stone risk profile (mg/mg)
1	20/M	History of tuber-culosis	Right percutaneous nephrosto-lithotomy for 22 mm stone	^CaOx monohydrate, 98%; Protein, 2%	Cit/Cr, 0.23 Ox/Cr, 0.02 Ca/Cr, 0.16 Ca/Cr, 0.23↑
2	13/F		Right percutaneous nephro-lithotomy for 16mm stone	CaOx monohydrate, 18%; CaOx dihydrate, 80%; Protein, 2%	No urine stone risk profile completed
3	15/F		Left ureteroscopy and laser lithotripsy for 5mm stone	CaOx monohydrate, 50%; CaOx dihydrate, 40%; CaPhos (hydroxyl form), 8%; Protein, 2%	Cit/Cr, 0.31 Ox/Cr, 0.03 Ca/Cr, 0.17
4	12/F	Ovarian cyst	Right ureteroscopy and laser lithotripsy for 4mm stone.	CaOx monohydrate, 55%; CaOx dihydrate, 15%; CaPhos (carbonate form), 8%; CaPhos (hydroxyl form), 20%; Protein, 2%	No urine stone risk profile completed
5	12/M	Seizures, treated with topiramate	Right ureteroscopy and laser lithotripsy for 11mm stone	Not completed.	Cit/Cr, 0.13↓ Ox/Cr, 0.04 Ca/Cr, 0.10 ^#^Ca/Cr, 0.23↑

^^^ different stone from same patient than stone used for microbiome sequencing.

^#^ random urine sample. Citrate/creatinine (Cit/Cr) normal is > 0.18 mg/mg. Oxalate to creatinine normal is < 0.1 mg/mg. Calcium/creatinine (Ca/Cr) normal is < 0.21 mg/mg.

### Bacteria and/or bacterial DNA is present in pediatric kidney stones

**Fig 2:**
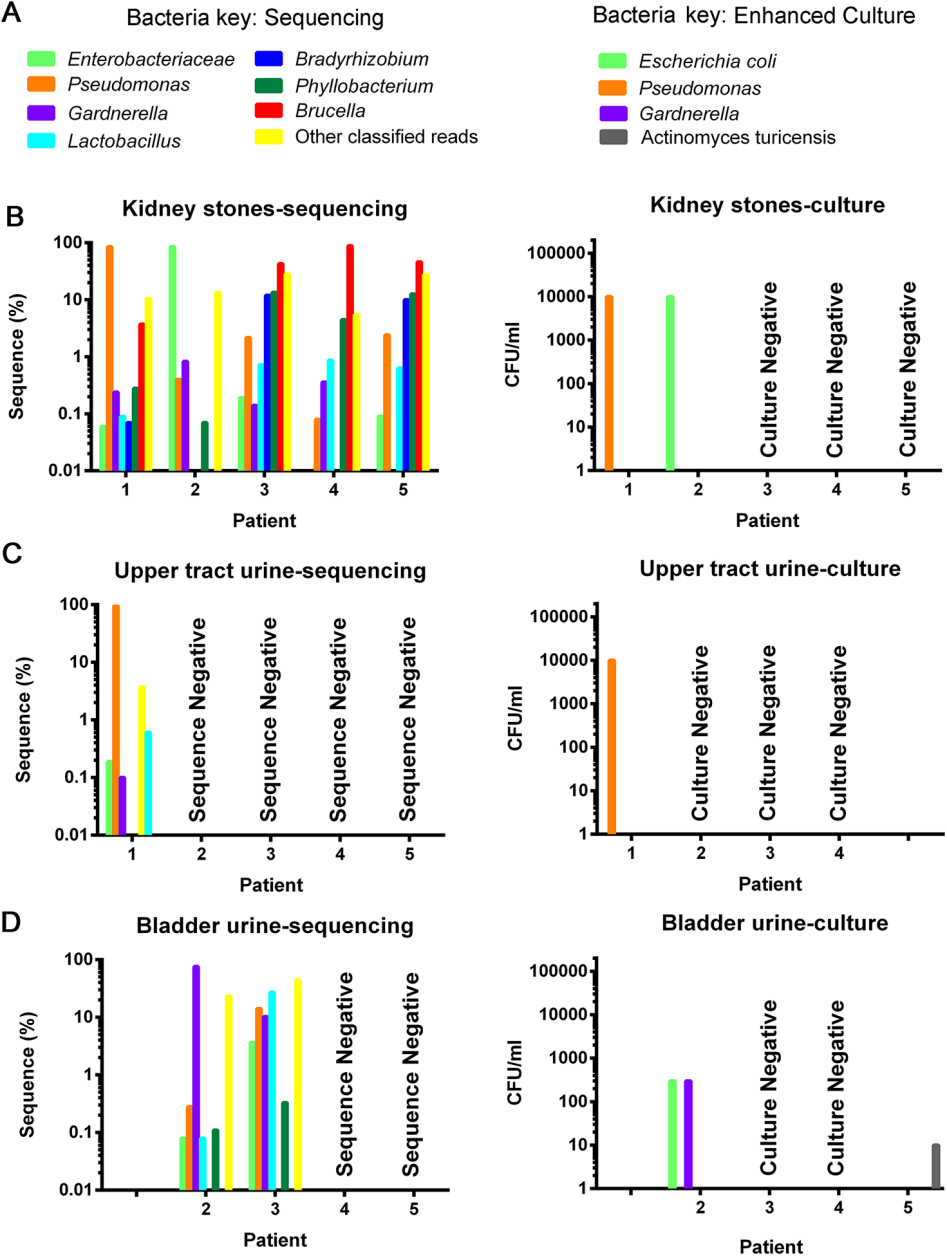
Kidney stones contain bacteria and/or DNA. (A) Bacteria key. (B) All stones contained multiple bacterial taxa on sequencing (left column) Bacteria identified included the family Enterobacteriaceae (which includes *E*. *coli*), and the genera *Pseudomonas*, *Gardnerella*, *Lactobacillus*, *Brucella*, *Phyllobacterium* and *Bradyrhizobium*. Bacteria were cultured (right column) from 2 stones and represented the most abundant bacteria identified by sequencing. (C) Bacteria were only sequenced and cultured from upper tract urine in 1 patient. (D) When detected in the bladder urine, the taxa were similar to those observed in the stones, although the ratios were often dissimilar. To allow low percentage organisms to be visualized, the data was graphed on a logarithmic Y-axis.

To guide murine studies and determine which, if any, bacteria are present in pediatric kidneys stones, we screened a limited number (5) of patients by EQUC and 16S rRNA gene sequencing. Bacterial DNA and live bacteria were detected in the kidney stones, upper tract (UT) urine, and bladder urine (Fig 2). From each of these 5 patients, we obtained kidney stones, bladder urine and/or upper tract urine for a total of 13 samples. Of these 13 samples, EQUC isolated viable bacteria from 5 samples: 2 out of 5 kidney stones, 1 out of 5 UT urine samples, and 2 out of 5 bladder urine samples. In contrast, 16S rRNA gene sequencing produced data from 8 out of 13 samples, including all 5 kidney stones. From these 8 samples, we obtained a total of 329,298 sequences. Excluding all sequences that were <0.01% of the reads in any sample, we identified 240 genera, 123 families, 66 orders, 42 classes, and 22 phyla. From 3 urine samples, we obtained a total of 188,099 sequences. Excluding all sequences that were <0.01% of the reads in any sample, we identified 108 genera, 70 families, 39 orders, 26 classes, and 12 phyla. From the 5 stones, we obtained a total of 158,848 sequences. Excluding all sequences that were <0.01% of the reads in any sample, we identified 201 genera, 109 families, 59 orders, 39 classes, and 19 phyla. All stones contained multiple bacterial taxa; however, *Pseudomonas* (85%) and Enterobacteriaceae (85%) dominated the stones from patients 1 and 2, respectively. These taxa were matched by EQUC, which cultured *Pseudomonas aeruginosa* from patient 1 and *Escherichia coli* from patient 2. Due to the lack of enough diversity in the V4 region, sequencing could not classify Enterobacteriaceae beyond the family level; however, this assignment matches the EQUC results, which identified *E*. *coli*. When detected in the bladder urines, the taxa were similar to those observed in the stones, although the ratios were often dissimilar. For example, whereas Enterobacteriaceae/*E*. *coli* dominated the stone microbiome of patient 2, *Gardnerella* was predominant in bladder urine. We selected uropathogenic *E*. *coli* (UPEC) for further *in vitro* and *in vivo* murine studies because experimental models for UPEC kidney infections are well established and there was evidence for Enterobacteriaceae/*E*. *coli* in human stones screened for by complementary EQUC and sequencing approaches.

### In vitro UPEC and CaOx culture

**Fig 3:**
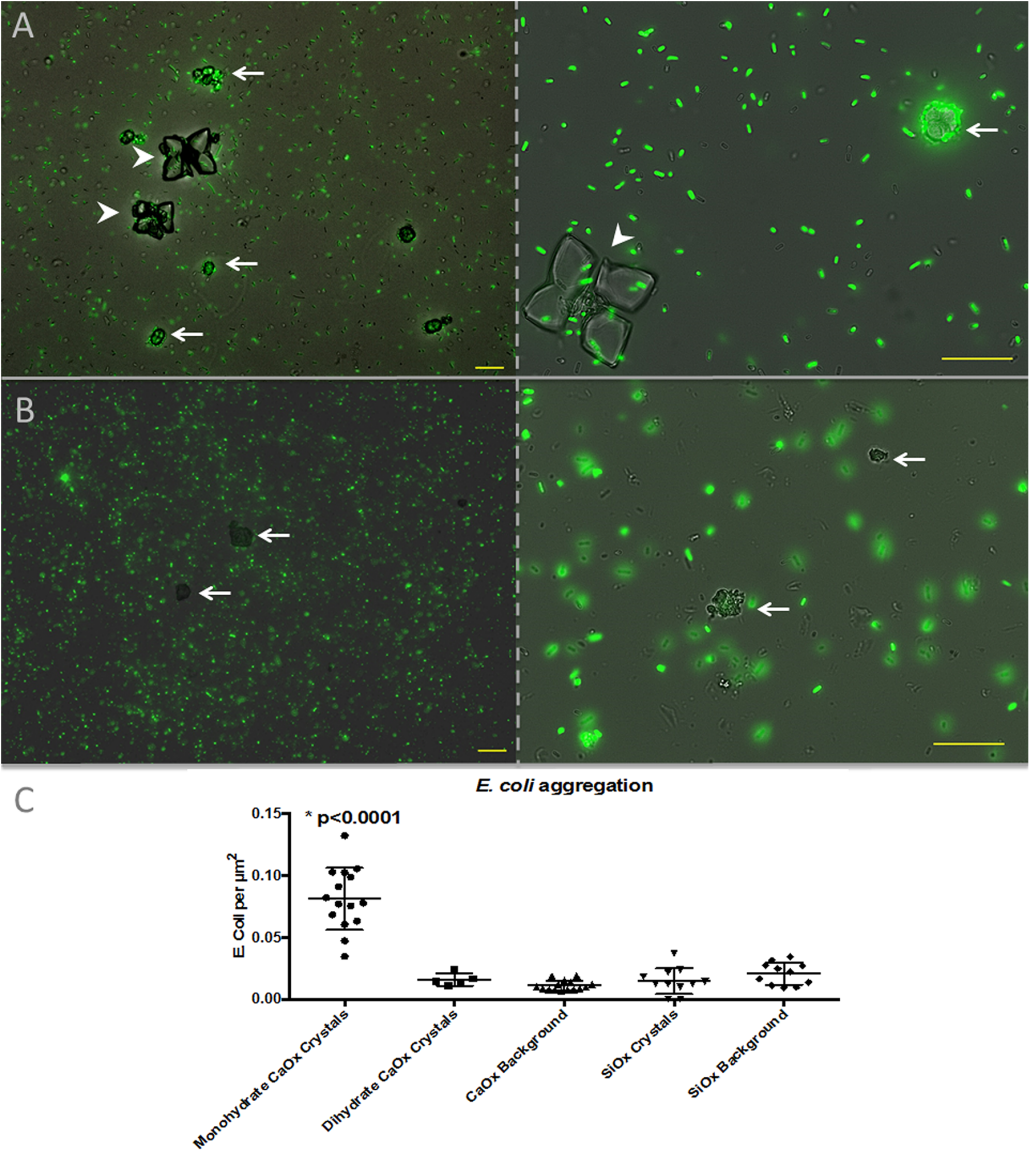
UPEC selectively aggregate around CaOx monohydrate crystals: following incubation with GFP labeled UPEC, bacteria (green) could be seen aggregating around CaOx monohydrate (A, arrows) but not CaOx dihydrate (A, arrowheads) or silicon dioxide (B, arrows) crystals. At 12 hours, significantly more bacteria per crystal surface area were seen with CaOx monohydrate crystals than with CaOx dihydrate crystals, silicon dioxide crystals or background. There were no other significant differences between groups. Magnification 40X right panels, 100X left panels Scale bars = 20 microns. Incubation time = 6 hours for left panels and 12 hours for right panels.

Uropathogenic *E*. *coli* (UPEC) aggregate on and around CaOx monohydrate crystals in significantly greater numbers compared to CaOx dihydrate and control silicon dioxide crystals (Fig 3). A time-lapse movie of UPEC aggregation on/around to CaOx is presented in S1 Video.

### Renal CaOx deposits increase the murine bacterial burden following UPEC inoculation

**Fig 4:**
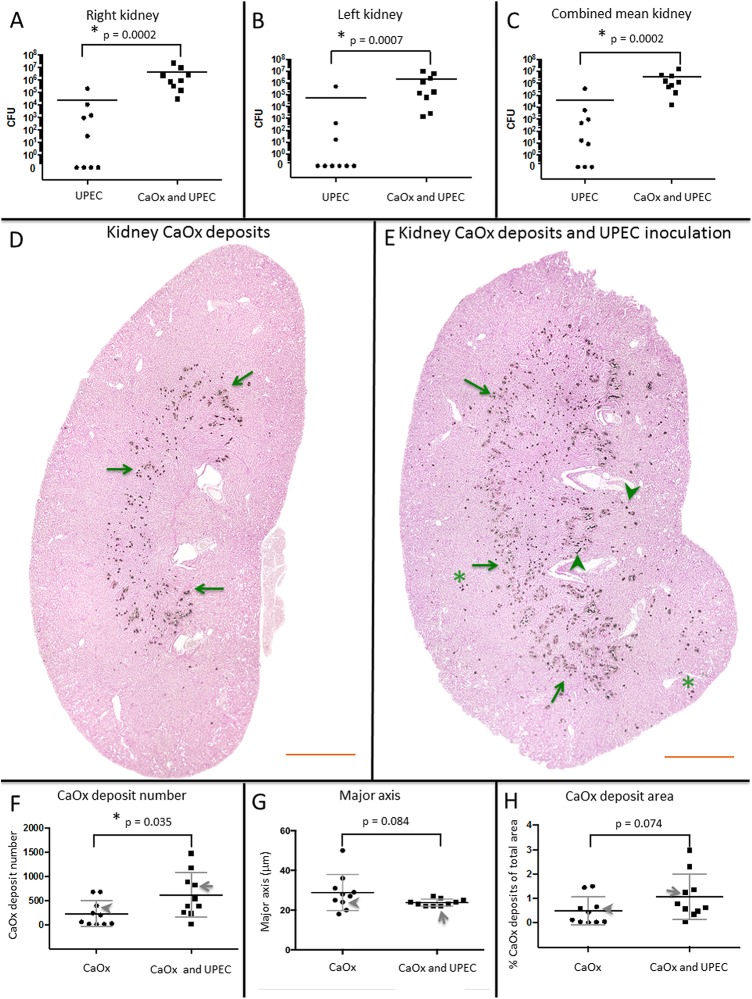
Bacteria increases the murine CaOx deposit burden. A. The mean right (A), left (B) and combined mean (C) kidney bacterial burdens were lower with UPEC inoculation alone compared to kidneys with CaOx deposits and UPEC inoculation at 56,157±1.68X10^5^ versus 2.14X10^6^±3.34X10^6^; 24,843±69,527 versus 4.50X10^6^±7.83X10^6^ and 40,500±1.12X10^5^ versus 5.28X10^6^±1.78X10^6^ respectively. To present on a log scale graph, but not during statistical analysis, 0 values were assigned a value of 0.01 (D). Following glyoxalate injection, CaOx deposits are seen around the corticomedullary junction (arrows) (E) Following UPEC inoculation and glyoxalate injection, an increased distribution and number of CaOx deposits (arrows) is noted, extending into the medulla (arrowheads). D-E, representative 4X magnification image stitches, background cropped for clarity, scale bar = 1000μm. (F) CaOx deposit number per mean 4X imaging stitch cross-section area was significantly higher in the CaOx deposits and UPEC inoculation group compared to kidney stones alone. There was a higher percentage of CaOx deposit/total kidney cross section area (H) with CaOx deposits and UPEC than CaOx deposits alone. The location on the scatterplot for the representative images (D) and (E) are indicated by an arrowhead and arrow respectively.

The bacterial burden was quantified 3 days after CaOx deposits induction (2 days post-UPEC inoculation). When both CaOx deposits and UPEC inoculation were present (n = 9), the bacterial burden was 181, 38 and 82-fold higher in the right kidney, left kidney and mean kidney, respectively compared to UPEC inoculation alone (n = 9);(Fig 4A–4C). Kidney cultures from saline control mice or mice inoculated with only CaOx demonstrated no bacterial growth at 24 hours.

### UPEC inoculation results in increased CaOx deposition

Mice with CaOx deposits and UPEC inoculation (n = 10) had a significantly higher number of CaOx deposits per mean 4X image stitch cross section than mice inoculated with CaOx alone (n = 10); (Fig 4D–4F). (Fig 4G and 4H). Renal CaOx deposits were not seen in the saline controls or in mice with UPEC inoculation alone (data not shown).

### CaOx deposits induce a murine renal innate immune response

**Fig 5:**
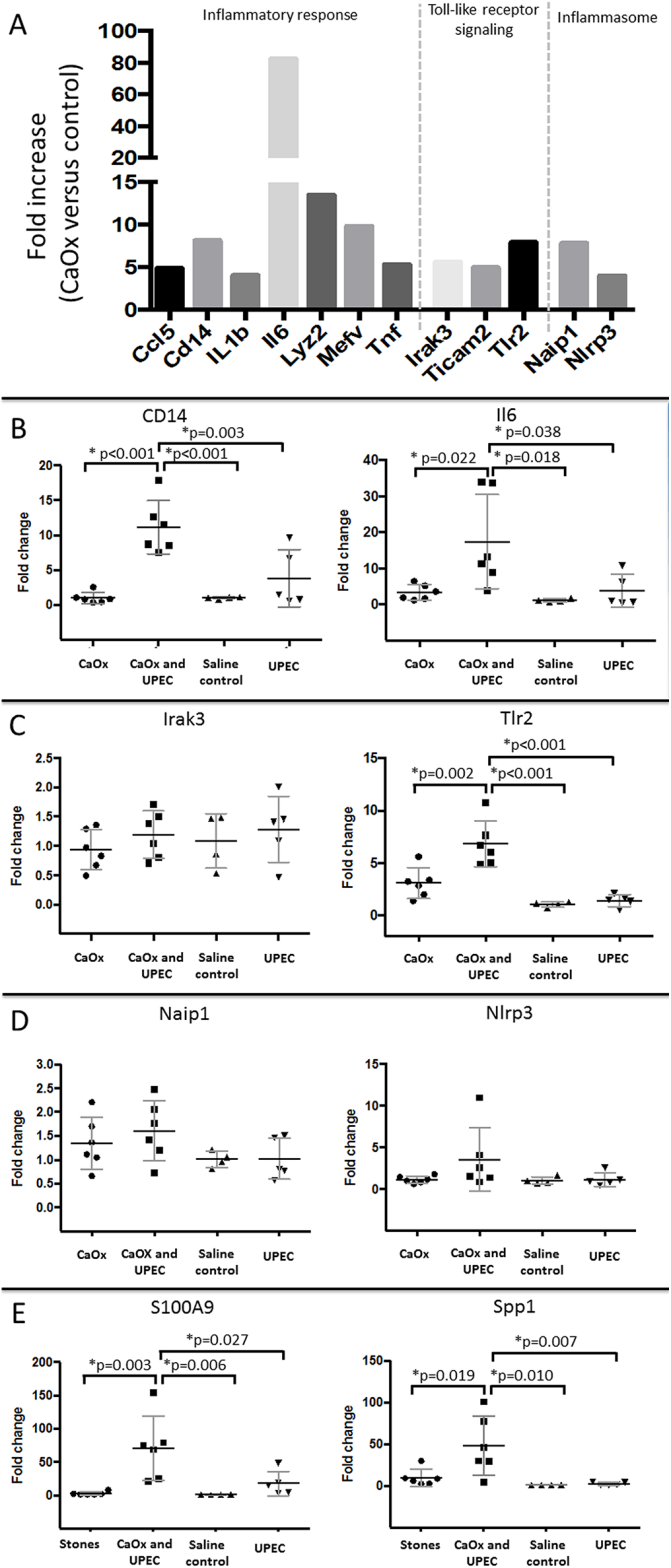
PCR results. (A) At peak murine CaOx deposit formation (6 days), the RT^2^ Bacterial Response Array revealed that 12/83 genes were significantly up-regulated > 4 fold. These genes included the inflammatory genes Chemokine (C-C motif) ligand 5 (*Ccl5*), Cluster of differentiation 14 (Cd14), Interleukin 1β (*Il1b*), Interleukin 6 (*Il6*), Lysozyme 2 (*Lyz2*), Mediterranean fever (*Mefv*), and Tumor necrosis factor (*Tnf*); the toll-like signaling genes, Interleukin–1 receptor-associated kinase 3 (*Irak3*) Toll-like receptor adaptor molecule 1 (*Ticam1*) and Toll-like receptor 2 (Tlr2) along with the inflammasome genes NLR family, apoptosis inhibitory protein 1 (*Naip*1) and NOD-like receptor family, pyrin domain containing 3 (*Nlrp3*). (B) In female C57Bl/6 mice, targeted RT-PCR revealed that at day 3 the inflammatory genes *CD14* and *Il6* are not increased in UPEC alone inoculated mice or CaOx alone inoculated mice compared to saline control, but are when both CaOx deposits and UPEC inoculation are present. (C) Toll-like signaling genes: *Tlr2* followed a similar pattern to the inflammatory genes while Irak3 was not increased at day 3. (D) The inflammasome genes were not up-regulated at 3 days in any of the groups (E) Stone matrix protein inner core components were markedly up-regulated, but only when both CaOx deposits and UPEC inoculation were present.

To identify innate immune genes with increased expression in response to CaOx deposits, we performed the RT^2^ Antibacterial Response Array. CaOx deposits induced a robust innate immune response at peak (6th day) CaOx deposits formation. Twelve of 84 genes were upregulated (>4-fold, P < 0.01) in mice with kidney CaOx deposits (n = 6) compared to saline controls (n = 6) (Fig 5A). The upregulated genes were involved with inflammation, toll-like receptor signaling and inflammasome formation. A table outlining the 84 studied genes, heat map and complete results are presented in S1 Table, S1 Fig and S1 File respectively.

### UPEC and CaOx together potentiate the murine renal innate immune response

To determine whether UPEC alters the CaOx induced innate immune response, we evaluated expression of innate immune genes in mice with UPEC inoculation alone (n = 5), kidney stones alone (n = 6), combined UPEC and CaOx inoculation (n = 6), and saline controls (n = 4). We also evaluated the expression of Osteopontin (Spp1) and Calgranulin B (S100A9), since they encode proteins present in the inner core of CaOx stones (15). No significant differences in inflammatory response, toll-like receptor signaling, inflammasome genes or stone matrix protein inner core genes were found with UPEC inoculation only, CaOx inoculation only or saline control mice. However, when mice were inoculated with both CaOx and UPEC, 2/2 inflammatory genes, 1/2 toll-like receptor gene and most notably 2/2 stone matrix protein genes were significantly elevated (Fig 5B–5D).

## Discussion

**Fig 6:**
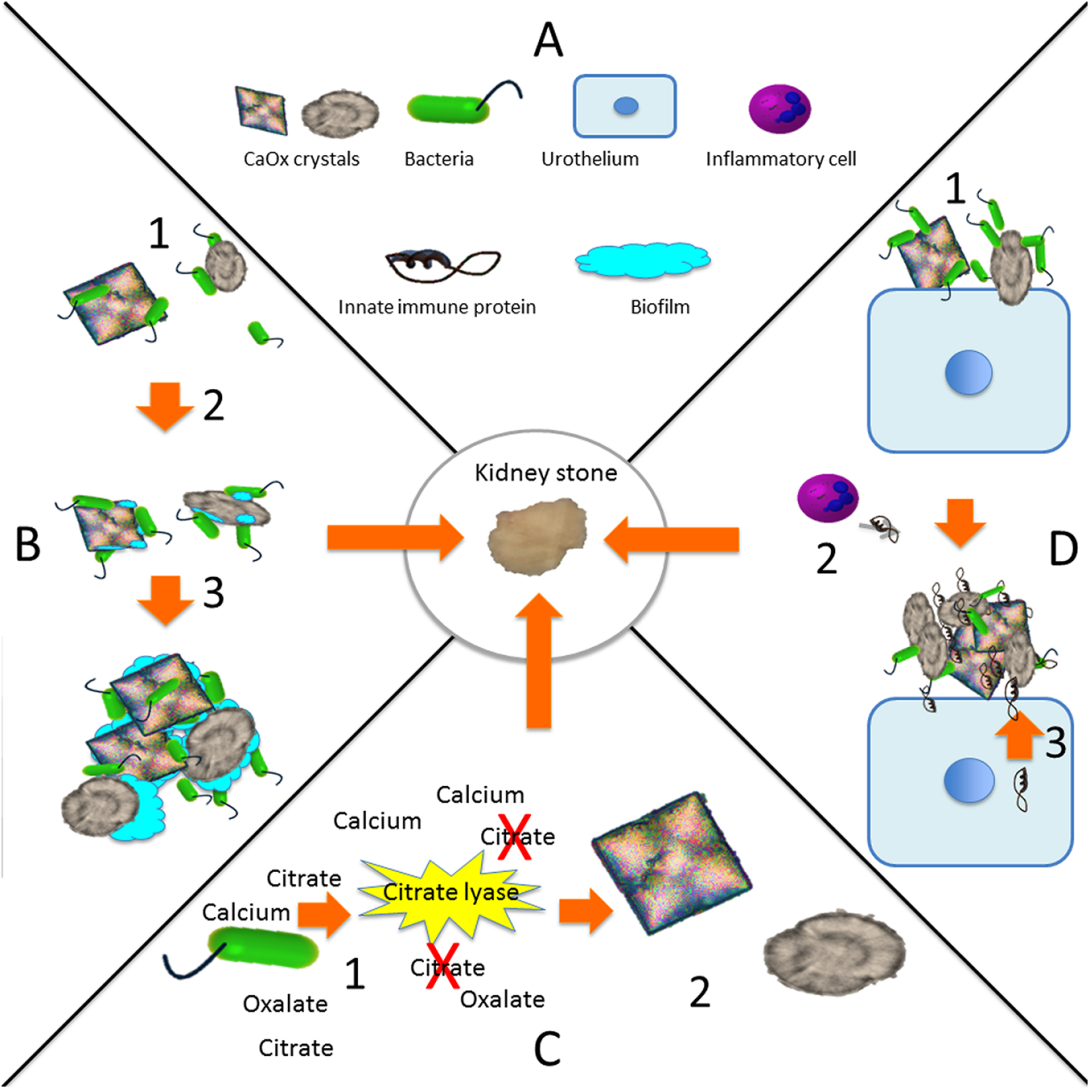
Speculated mechanisms for bacterial contribution to CaOx stones. (A) Figure key. (B) Bacteria bind to CaOx crystals that may provide a nidus for pyelonephritis or remain persist in a subclinical state (1) and bacterial communities form a biofilm (2). The biofilm results in crystal aggregation (3). (C) The bacterial enzymes citrate lyase splits citrate resulting in increased CaOx supersaturation (1). CaOx crystals form providing a key element of lithogenesis. (D) Bacteria bind to the urothelium (1) that results in secretion of innate immune proteins from recruited inflammatory cells (2) and the urothelium (3) The innate immune proteins are incorporated as stone matrix proteins.

We hypothesize that bacteria and renal CaOx deposits potentiate individually induced nephropathy (Fig 6). Urine is not always sterile and contains a microbiome[22, 23]. Thus, dysbiosis may create a urinary environment conducive to kidney stone formation; conversely, CaOx crystals or deposits may form a nidus for bacteria, increasing UTI risk. In support of our hypothesis, we have demonstrated that human kidney stones have a microbiome (Fig 2), UPEC aggregate around CaOx monohydrate crystals (Fig 3), renal CaOx deposits potentiate murine pyelonephritis (Fig 4A–4C). UPEC inoculation of the murine kidney increases calcium oxalate deposition (Fig 4D–4H) and induces local expression of innate immune proteins (Fig 5) associated with the stone inner core.

Children with idiopathic hypercalciuria have increased rates of urinary tract infections [24, 25]. Furthermore, patients with non-struvite kidney stones often have positive urine cultures [8]. We were able to detect bacteria in human kidney stones but not in upper tract urine; this suggests that the CaOx stones represent an area of concentrated bacteria. Our murine studies provide the first experimental evidence that renal CaOx deposits increase pyelonephritis susceptibility Despite the frequency of kidney stones and UTIs, the biologic, diagnostic, and therapeutic relevance of their association remains largely unknown. Determining whether treatment of hypercalciuria and kidney stones reduces UTI risk remains to be determined. Additionally, a more extensive evaluation of CaOx crystal and *UPEC* adhesion forces with atomic force microscopy warrants consideration.

The identification of bacteria in CaOx kidney stones raises the possibility that bacteria or bacterial biofilms are components of CaOx stone formation (Fig 6B) as they are in struvite stones[4]. Four out of 5 (80%) of the kidney stones were positive for Enterobacteriaceae family by sequencing. EQUC identified *E*. *coli*, a major member of family Enterobacteriaceae in one of the three kidney stones. These complementary methods for bacterial identification indicate that Enterobacteriaceae may be associated with pediatric kidney stones. Since the positive cultures were from large kidney stones (Table 1, Fig 2) it is possible that EQUC may not be sensitive in smaller kidney stones with smaller bacterial loads. Additionally, sequencing was positive for *Pseudomonas* and *Gardnerella* in 5/5 and 4/5 stones, respectively; both species have been detected previously in adult CaOx stones. Sequencing identified bacteria in 100% of stones, well above the 19–32% positive CaOx culture rate previously reported [7, 8]. Whenever EQUC identified a bacterium, sequencing also identified it. In contrast, sequencing identified many more bacteria than did EQUC. The increased sensitivity of sequencing relative to culture may be due to the inability of some bacteria to be readily cultured, the ability of sequencing to detect and identify bacteria that are not viable or culturable, and the ability of sequencing to detect smaller numbers of bacteria. This increased likelihood of finding bacteria with sequencing compared to culture correlates with what has been described previously for the urinary microbiome [13].

We are not aware of prior reports of *Bradyrizobium* in urine or kidney stones, but it has been identified elsewhere in the human microbiome [26]. Phyllobacterium is an environmental organism [27]. *Brucella* does not appear to be a contaminating organism because it was not present in controls including sterile PBS and the bullet blender beads. It also was not found in the upper tract urine. Furthermore *Brucella* is not an environmental organism or found spontaneously in mice but may be found in livestock and unpasteurized milk, which the investigating facilities do not study[28]. Our high throughput sequencing uses the V4 hypervariable region of the 16S rRNA gene and is therefore only able to classify to the genus level. Therefore, what the sequence analysis identified as *Brucella* could represent an organism that has a similar V4 region such as *Ochrobactrum*[29]. *Ochrobactrum* has been identified in human urine and we submit that our findings may demonstrate that it may also be found in kidney stones[30].

With the exception of patient 1, the upper tract urine was culture-negative and sequence-negative. Therefore it is unlikely that bacteria DNA from the surrounding upper tract urine contaminated the kidney stone. This discrepancy in bacterial loads between the upper tract urine and kidney stones could be explained by pre-operative prophylactic antibiotics that patients received, which might have had time to sterilize the upper tract urine but not reach the inner stone or the bladder urine. Alternatively, the upper tract with higher flow may not be conducive to bacterial growth compared to the relative urine stasis in the bladder. Additionally, the CaOx surface characteristics may be more prone to bacterial growth or colonization than urine or urine epithelial cells.

We demonstrated that *E*. *coli* aggregates around CaOx monohydrate crystals compared to CaOx dihydrate crystals and control silicon dioxide crystals. Bacterial adhesion to crystals is complex because biological properties very based on crystalline form[31]. Future studies will include direct evaluation of adhesive forces between a wide variety of crystals and bacteria with atomic force microscopy[32]. Our findings build upon the findings by Chutipongtanate and co-workers who demonstrated that bacteria including *E*. *coli* interact with CaOx crystals[33]. Specifically they demonstrated that intact viable, but not dead, bacteria enlarged CaOx aggregates[33].

Traditionally, supersaturation of CaOx has been considered the primary mechanism for stone formation. Bacteria in kidney stones or urine may alter urine supersaturation (Fig 6C). Prior research demonstrated that culture positive urine samples have lower citrate levels than culture-negative samples and lower bacterial utilization of citrate via citrate lyase [34, 35]. The correlation of urine chemistries with the urine and kidney stone microbiome was beyond the scope of this project, but remains an area for future research.

Human kidney stones are increasingly recognized to have an inflammatory component [36–38]. The kidney stone matrix is predominated by inflammatory proteins. It has been shown that CaOx crystals induce an inflammatory response through dendritic cell secretion of Il–1β, inflammasomes are involved in kidney stone pathogenesis and urine Il–6 levels are increased in patients with kidney stones [39–42]. Furthermore, the innate immune proteins Osteopontin (Spp1) and Calgranulin B (S100A9) have consistently been identified in the inner core of CaOx deposits [43]. A potential mechanism for kidney stone formation consists of bacteria/CaOx induction of innate immune proteins that are incorporated into the kidney stone matrix inner core, leading to progression from crystalluria to stone formation (Fig 6D). The finding that most inflammatory mediators were not significantly elevated in oxalate loaded mice versus saline controls or UPEC alone indicated that the this immune response is not due to oxalate poisoning but represent synergy between CaOx and UPEC (Fig 5). A similar mechanism occurs in extra-renal calcifications. Specifically, bacteria are present in vascular calcifications and innate immune recognition of bacteria accelerates atherosclerosis [44, 45]. At an early time point in murine CaOx induced disease, an oxalate load or UPEC inoculation individually did not significantly increase the innate immune response, whereas the combination of CaOx deposit and UPEC inoculation increased both the innate immune response, most importantly the stone inner core matrix protein gene expression and the renal CaOx deposit burden.

## Conclusions

Kidney stones have a microbiome that includes *Enterobacteriaceae*. *E*. *coli* aggregate around CaOx monohydrate crystals, and murine UPEC inoculation is associated with increased renal CaOx deposition along with the innate immune response, including increased expression of stone matrix proteins, during experimental murine CaOx nephropathy. Further renal CaOx depositions increase the bacterial burden during murine pyelonephritis. Perhaps kidney stones and/or CaOx crystalluria represents a reversible risk factor that should be screened for in patients with recurrent pyelonephritis. Limitations of this study include lack of documentation of stone composition in the kidney stone of one patient and the previously mentioned limitations in bacterial DNA sequencing technology. Future studies will be needed to confirm whether low abundance organisms are passive environmental contaminants or directly involved in lithogenesis. Additionally murine renal CaOx models have key differences from human kidney stone disease. We studied human urolithiasis, *in vitro* CaOx crystals and murine CaOx deposits under the broad umbrella of CaOx disease. CaOx deposition in mice occurs acutely and is located mostly in the interstitial space. In humans CaOx deposition is mostly tubular and occurs over a long period of time. Because of these key differences, our murine findings will need to be validated in human patients or an animal model, when one is available that closely approximates human urolithiasis, to determine whether bacteria have any relevance in human kidney stone disease. Whether bacteria are essential for CaOx kidney stone formation, a disease-modifying factor or largely an uninvolved association remains to be determined. However, this study provides proof-of-concept that bacteria can worsen the CaOx kidney deposit disease course and vice versa. Our screen of 5 kidney stones for bacteria was meant to guide our murine studies, clearly sequencing of a much larger cohort of patients is needed to characterize the kidney stone microbiome and determine if the bacteria we identified are frequently present in other cohorts. Future studies will also be needed to determine mechanisms responsible for the association and/or interaction between bacteria and calcium oxalate disease.

## Supporting Information

S1 Fig (A) Pizzolato stain of a 4X cross-section image stitch (upper panel) in which the CaOx deposits are stained black. A zoom view (lower panel) demonstrates individual CaOx deposits (B) The “dynamic cell count” feature of the BZ900 microscope software identifies and outlines the CaOx deposits (yellow) in the 4X image stitch (upper panel) with the outlines of individual software-identified CaOx deposits visible in zoom view (lower panel). Subsequently the software records the number, major axis and area of the kidney CaOx deposits.
(TIF)

S2 Fig A heatmap (upper panel) and table (lower panel) of the RT^2^ Profiler Antibacterial Response Array results. (TIF)

S1 File Complete RT^2^ profiler Antibacterial Response Array results. (PDF)

S1 Table A table of all of the genes in the RT^2^ Profiler Antibacterial Response Array. (DOCX)

S1 Video Time-lapse movie of GFP labeled *E*. *coli* (green) incubation with CaOx crystals. At around 6 hours *E*. *coli* begins to surround dumbbell shaped CaOx monohydrate crystals with a similar process occurring to tetragonal di-pyramid CaOx dihydrate crystals several hours later. Images were taken at 20X magnification and 20°Celsius.
(MOV)
