# Supplementary figures and images for: The Interaction between Enterobacteriaceae and Calcium Oxalate Deposits
Source: PLoS One. 2015 Oct 8;10(10):e0139575. doi: 10.1371/journal.pone.0139575 (PMC4598009; doi:10.1371/journal.pone.0139575)

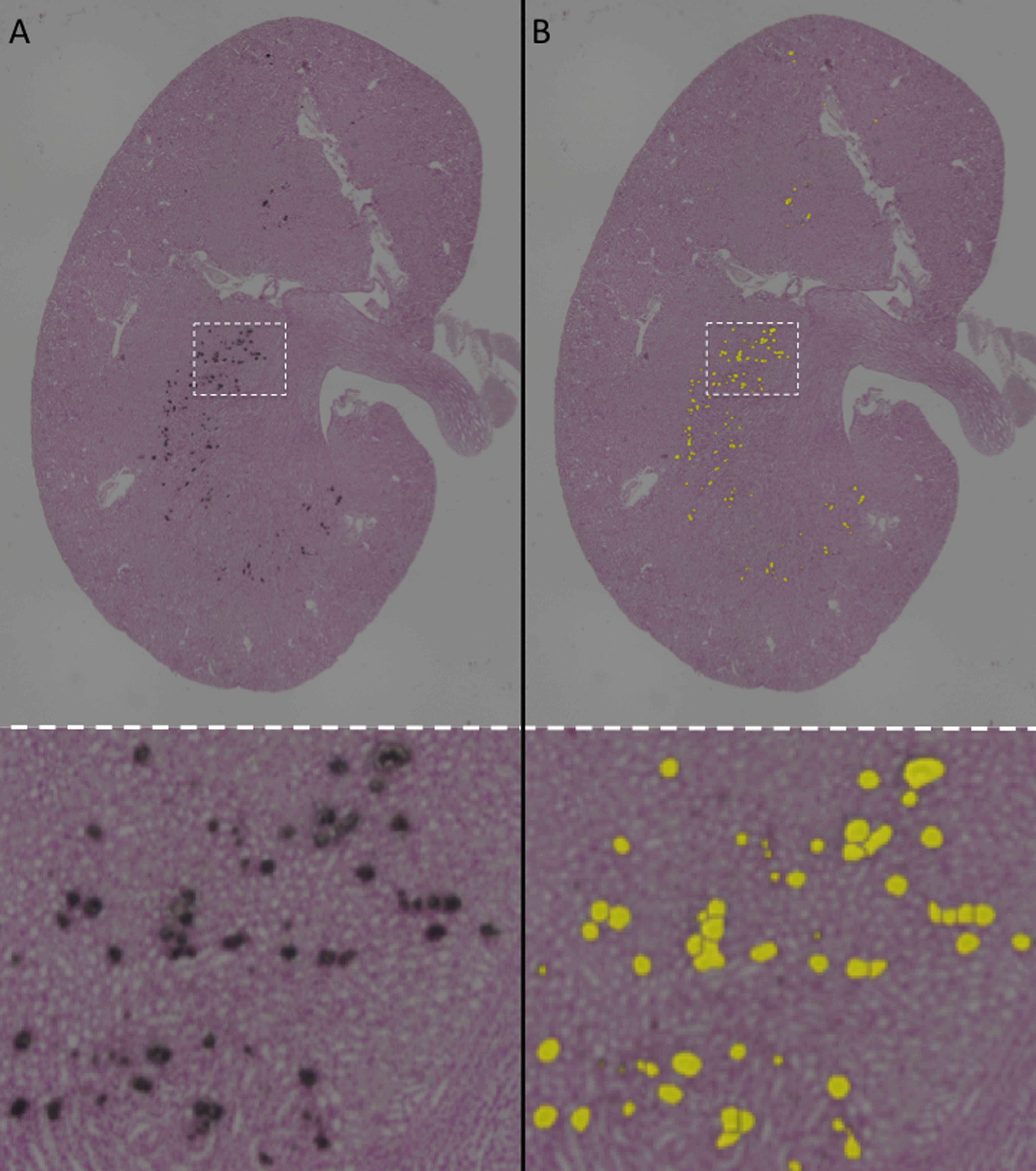

Supplement: S1 Fig — Subsequently the software records the number, major axis and area of the kidney CaOx deposits. (TIF) [file pone.0139575.s001.tif]

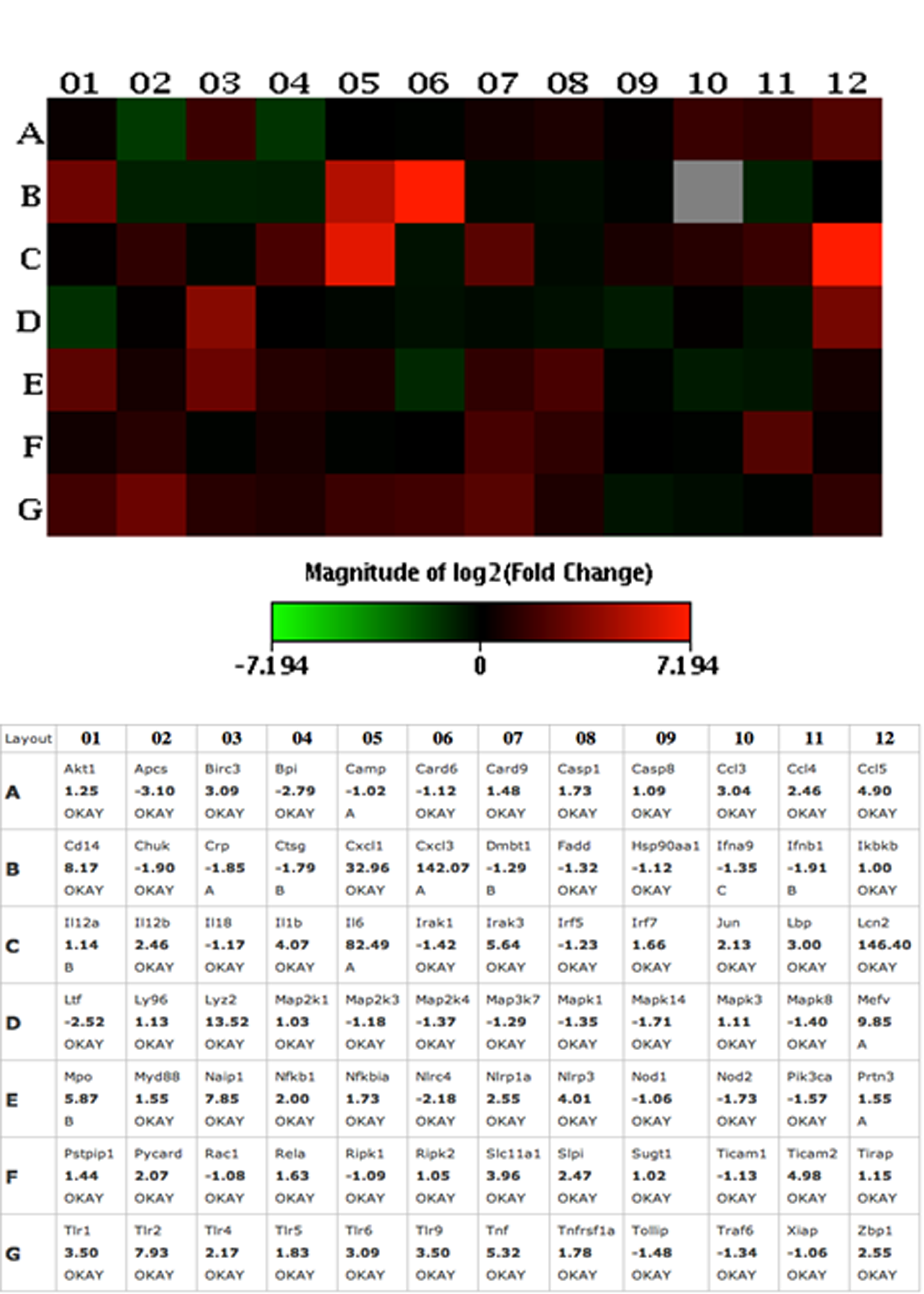

Supplement: S2 Fig — (TIF) [file pone.0139575.s002.tif]
