# Supplementary material for: The Interaction between Enterobacteriaceae and Calcium Oxalate Deposits
Source: PLoS One. 2015 Oct 8;10(10):e0139575. doi: 10.1371/journal.pone.0139575 (PMC4598009; doi:10.1371/journal.pone.0139575)
Supplement: S1 File — (PDF) [file pone.0139575.s003.pdf]

		AVG Ct		Standard Deviation	
Position	Symbol	Control Grc	Kidney stones	Control Group	Kidney stones
A01	Akt1	20.76	20.02	0.29708	0.428754
A02	Apcs	25.86	27.06	0.235747	0.733233
A03	Birc3	23.21	21.15	0.177501	0.416773
A04	Bpi	32.66	33.72	1.216202	1.380192
A05	Camp	30.26	29.86	0.828792	0.466819
A06	Card6	23.76	23.5	0.145705	0.462363
A07	Card9	26.4	25.41	0.252382	0.28376
A08	Casp1	25.73	24.52	0.183811	0.307197
A09	Casp8	20.74	20.19	0.146697	0.377796
A10	Ccl3	27.91	25.87	0.70045	0.50013
A11	Ccl4	27.24	25.51	0.470592	0.460728
A12	Ccl5	24.45	21.73	0.387969	0.390704
B01	Cd14	23.66	20.2	0.186726	0.436715
B02	Chuk	17.78	18.28	0.098928	0.198796
B03	Crp	29.89	30.35	0.693669	0.737869
B04	Ctsg	33.63	34.04	1.40209	0.98814
B05	Cxcl1	28.02	22.54	0.983234	0.703974
B06	Cxcl3	34.14	26.56	0.76212	0.753903
B07	Dmbt1	34.7	34.64	0.730764	0.809457
B08	Fadd	21.73	21.71	0.223607	0.337609
B09	Hsp90aa1	17.38	17.11	0.343827	0.435798
B10	Ifna9	35	35	0	0
B11	Ifnb1	30.58	31.09	0.399433	0.567829
B12	Ikbkb	21.13	20.7	0.296743	0.483084
C01	Il12a	31.82	31.21	0.469698	0.739865
C02	Il12b	29.72	27.99	0.814387	0.485438
C03	Il18	23.89	23.69	0.170529	0.416089
C04	Il1b	26.3	23.85	0.227508	0.708957
C05	Il6	32.14	25.34	0.971358	0.584491
C06	Irak1	19.97	20.05	0.141374	0.207557
C07	Irak3	26.96	24.03	3.952896	0.606902
C08	Irf5	22.07	21.95	0.337026	0.520701
C09	Irf7	22.68	21.52	0.302787	0.423887
C10	Jun	21.06	19.54	0.590844	0.560286
C11	Lbp	25.26	23.25	0.352784	0.61524
C12	Lcn2	24.71	17.09	0.283125	0.970577
D01	Ltf	27.73	28.64	0.334285	0.180887
D02	Ly96	23.16	22.55	0.272592	0.25446
D03	Lyz2	21.86	17.68	0.278041	0.544858
D04	Map2k1	20.71	20.24	0.311897	0.451187
D05	Map2k3	20.51	20.32	0.215932	0.40906
D06	Map2k4	20.96	20.99	0.201668	0.46838
D07	Map3k7	20.81	20.74	0.251767	0.450744
D08	Mapk1	18.92	18.92	0.265481	0.398309
D09	Mapk14	19.75	20.1	0.225802	0.376125
D10	Mapk3	20.77	20.19	0.298239	0.477022
D11	Mapk8	21.92	21.97	0.166333	0.339338

D12	Mefv	31.24	27.51	0.648704	0.562165
E01	Mpo	34.93	31.95	0.175547	1.395733
E02	Myd88	25.17	24.11	0.344248	0.678174
E03	Naip1	29.31	25.91	0.395255	1.270146
E04	Nfkb1	21.91	20.48	0.229492	0.540897
E05	Nfkbia	20.41	19.19	0.538628	0.516459
E06	Nlrc4	23.68	24.38	0.111788	0.55908
E07	Nlrp1a	28.4	26.62	0.413267	0.683827
E08	Nlrp3	28.76	26.33	0.375908	0.730664
E09	Nod1	23.45	23.11	0.22151	0.410828
E10	Nod2	25.73	26.09	0.275808	0.339588
E11	Pik3ca	20.22	20.44	0.184246	0.315706
E12	Prtn3	30.18	29.12	0.288975	0.273276
F01	Pstpip1	24.95	24	1.947639	0.371039
F02	Pycard	22.7	21.23	0.164762	0.416389
F03	Rac1	17.84	17.53	0.206785	0.173147
F04	Rela	22.48	21.35	0.248871	0.435052
F05	Ripk1	22.41	22.11	0.181485	0.418294
F06	Ripk2	24.02	23.52	0.1747	0.28332
F07	Slc11a1	25.76	23.35	0.199566	0.630198
F08	Slpi	27.9	26.17	1.187485	0.316417
F09	Sugt1	20.24	19.78	0.243454	0.371443
F10	Ticam1	22.55	22.3	0.365646	0.425029
F11	Ticam2	27.67	24.93	0.325003	0.515868
F12	Tirap	24.25	23.61	0.305614	0.486549
G01	Tlr1	26.37	24.13	0.361096	0.340955
G02	Tlr2	26.03	22.61	0.301159	0.901565
G03	Tlr4	24.68	23.13	0.250333	0.504698
G04	Tlr5	28.24	26.94	0.481557	0.444994
G05	Tlr6	27.71	25.66	0.276381	0.474689
G06	Tlr9	25.88	23.64	0.35259	0.446464
G07	Tnf	28.73	25.89	0.74153	0.588583
G08	Tnfrsf1a	20.33	19.07	0.146071	0.430198
G09	Tollip	20.41	20.55	0.285494	0.416857
G10	Traf6	21.11	21.09	0.190026	0.382858
G11	Xiap	21.58	21.24	0.169519	0.453189
G12	Zbp1	25.11	23.33	0.314685	0.426063
H01	Actb	15.58	14.76	0.27941	0.549026
H02	B2m	16.31	15.03	0.260998	0.487524
H03	Gapdh	15.06	15.23	0.266808	0.359819
H04	Gusb	21.14	20.97	0.349595	0.448141
H05	Hsp90ab1	15.85	15.8	0.202262	0.355711
H06	MGDC	35	35	0	0
H07	RTC	19.96	20.05	0.155177	0.279857
H08	RTC	19.92	20.01	0.16943	0.252844
H09	RTC	19.96	20.03	0.173051	0.265838
H10	PPC	17.46	17.49	0.040332	0.193391
H11	PPC	17.45	17.44	0.032863	0.05933
H12	PPC	17.43	17.38	0.073485	0.067971

		Up-Down Regulation (comparing to control group)	
		Kidney stones	
Position	Symbol	Fold Regula	Comments
A01	Akt1	1.2461	OKAY
A02	Apcs	-3.1034	OKAY
A03	Birc3	3.0918	OKAY
A04	Bpi	-2.7912	OKAY
A05	Camp	-1.0172	A
A06	Card6	-1.1244	OKAY
A07	Card9	1.4758	OKAY
A08	Casp1	1.7256	OKAY
A09	Casp8	1.0851	OKAY
A10	Ccl3	3.0373	OKAY
A11	Ccl4	2.4648	OKAY
A12	Ccl5	4.8967	OKAY
B01	Cd14	8.1707	OKAY
B02	Chuk	-1.8959	OKAY
B03	Crp	-1.8487	A
B04	Ctsg	-1.7874	B
B05	Cxcl1	32.9636	OKAY
B06	Cxcl3	142.0708	A
B07	Dmbt1	-1.2875	B
B08	Fadd	-1.3234	OKAY
B09	Hsp90aa1	-1.1187	OKAY
B10	Ifna9	-1.3456	C
B11	Ifnb1	-1.9064	B
B12	Ikbkb	1.001	OKAY
C01	Il12a	1.1356	B
C02	Il12b	2.4597	OKAY
C03	Il18	-1.1746	OKAY
C04	Il1b	4.0665	OKAY
C05	Il6	82.4892	A
C06	Irak1	-1.4151	OKAY
C07	Irak3	5.6352	OKAY
C08	Irf5	-1.2319	OKAY
C09	Irf7	1.6561	OKAY
C10	Jun	2.126	OKAY
C11	Lbp	3.001	OKAY
C12	Lcn2	146.4029	OKAY
D01	Ltf	-2.519	OKAY
D02	Ly96	1.1317	OKAY
D03	Lyz2	13.521	OKAY
D04	Map2k1	1.028	OKAY
D05	Map2k3	-1.179	OKAY
D06	Map2k4	-1.3748	OKAY
D07	Map3k7	-1.2866	OKAY
D08	Mapk1	-1.3456	OKAY

D09	Mapk14	-1.7134	OKAY
D10	Mapk3	1.1084	OKAY
D11	Mapk8	-1.3962	OKAY
D12	Mefv	9.8455	A
E01	Mpo	5.865	B
E02	Myd88	1.5481	OKAY
E03	Naip1	7.8524	OKAY
E04	Nfkb1	1.9951	OKAY
E05	Nfkbia	1.7264	OKAY
E06	Nlrc4	-2.1803	OKAY
E07	Nlrp1a	2.547	OKAY
E08	Nlrp3	4.0087	OKAY
E09	Nod1	-1.0626	OKAY
E10	Nod2	-1.7305	OKAY
E11	Pik3ca	-1.5658	OKAY
E12	Prtn3	1.5502	A
F01	Pstpip1	1.4361	OKAY
F02	Pycard	2.0693	OKAY
F03	Rac1	-1.0824	OKAY
F04	Rela	1.6262	OKAY
F05	Ripk1	-1.0902	OKAY
F06	Ripk2	1.0525	OKAY
F07	Slc11a1	3.959	OKAY
F08	Slpi	2.4688	OKAY
F09	Sugt1	1.0173	OKAY
F10	Ticam1	-1.1328	OKAY
F11	Ticam2	4.9834	OKAY
F12	Tirap	1.1549	OKAY
G01	Tlr1	3.4987	OKAY
G02	Tlr2	7.9345	OKAY
G03	Tlr4	2.1742	OKAY
G04	Tlr5	1.8346	OKAY
G05	Tlr6	3.0933	OKAY
G06	Tlr9	3.5011	OKAY
G07	Tnf	5.3189	OKAY
G08	Tnfrsf1a	1.7803	OKAY
G09	Tollip	-1.4813	OKAY
G10	Traf6	-1.3353	OKAY
G11	Xiap	-1.0613	OKAY
G12	Zbp1	2.5511	OKAY
H01	Actb	1.3111	OKAY
H02	B2m	1.7998	OKAY
H03	Gapdh	-1.5194	OKAY
H04	Gusb	-1.1941	OKAY
H05	Hsp90ab1	-1.3006	OKAY
H06	MGDC	-1.3456	C
H07	RTC	-1.4302	OKAY
H08	RTC	-1.4249	OKAY
H09	RTC	-1.4072	OKAY

H10	PPC	-1.377	OKAY
H11	PPC	-1.3344	OKAY
H12	PPC	-1.3015	OKAY

		**p-value (comparing to control group)**
Position	Symbol	Kidney stones
A01	Akt1	0.021149
A02	Apcs	0.000009
A03	Birc3	0
A04	Bpi	0.039051
A05	Camp	0.759651
A06	Card6	0.547259
A07	Card9	0.009481
A08	Casp1	0.000884
A09	Casp8	0.263556
A10	Ccl3	0.001773
A11	Ccl4	0.000148
A12	Ccl5	0.000063
B01	Cd14	0
B02	Chuk	0.000897
B03	Crp	0.032714
B04	Ctsg	0.298294
B05	Cxcl1	0.000001
B06	Cxcl3	0.000009
B07	Dmbt1	0.664454
B08	Fadd	0.001706
B09	Hsp90aa1	0.227384
B10	Ifna9	0.073941
B11	Ifnb1	0.138077
B12	Ikbkb	0.934199
C01	Il12a	0.463384
C02	Il12b	0.000583
C03	Il18	0.012673
C04	Il1b	0.000107
C05	Il6	0.000007
C06	Irak1	0.00286
C07	Irak3	0.009074
C08	Irf5	0.037471
C09	Irf7	0.001619
C10	Jun	0.000541
C11	Lbp	0.000065
C12	Lcn2	0.072431
D01	Ltf	0.000261
D02	Ly96	0.099044
D03	Lyz2	0
D04	Map2k1	0.798168
D05	Map2k3	0.047252

D06	Map2k4	0.000792
D07	Map3k7	0.005031
D08	Mapk1	0.004072
D09	Mapk14	0.000055
D10	Mapk3	0.198127
D11	Mapk8	0.004163
D12	Mefv	0.000001
E01	Mpo	0.051424
E02	Myd88	0.001549
E03	Naip1	0.005997
E04	Nfkb1	0.000002
E05	Nfkbia	0.008648
E06	Nlrc4	0.000065
E07	Nlrp1a	0.000052
E08	Nlrp3	0.000018
E09	Nod1	0.569068
E10	Nod2	0.001635
E11	Pik3ca	0.000242
E12	Prtn3	0.088731
F01	Pstpip1	0.588986
F02	Pycard	0.000002
F03	Rac1	0.473933
F04	Rela	0.000004
F05	Ripk1	0.165585
F06	Ripk2	0.613671
F07	Slc11a1	0.000009
F08	Slpi	0.15921
F09	Sugt1	0.839689
F10	Ticam1	0.205792
F11	Ticam2	0.000001
F12	Tirap	0.182646
G01	Tlr1	0.000002
G02	Tlr2	0.000169
G03	Tlr4	0.000017
G04	Tlr5	0.013125
G05	Tlr6	0
G06	Tlr9	0
G07	Tnf	0.000012
G08	Tnfrsf1a	0.000001
G09	Tollip	0.00101
G10	Traf6	0.00062
G11	Xiap	0.398978
G12	Zbp1	0.000001
H01	Actb	0.00089
H02	B2m	0.000326
H03	Gapdh	0.000004
H04	Gusb	0.024147
H05	Hsp90ab1	0.000044
H06	MGDC	0.073941

H07	RTC	0.003596
H08	RTC	0.003925
H09	RTC	0.007792
H10	PPC	0.155461
H11	PPC	0.098228
H12	PPC	0.144039

		**Fold Change (comparing to control group)**		
		Kidney stones		
Position	Symbol	Fold Chang	95% CI	Comments
A01	Akt1	1.2461	(1.07, 1.43)	OKAY
A02	Apcs	0.3222	(0.15, 0.49)	OKAY
A03	Birc3	3.0918	(2.80, 3.39)	OKAY
A04	Bpi	0.3583	(0.04, 0.68)	OKAY
A05	Camp	0.9831	(0.36, 1.60)	A
A06	Card6	0.8893	(0.64, 1.13)	OKAY
A07	Card9	1.4758	(1.14, 1.81)	OKAY
A08	Casp1	1.7256	(1.36, 2.09)	OKAY
A09	Casp8	1.0851	(0.95, 1.22)	OKAY
A10	Ccl3	3.0373	(1.73, 4.35)	OKAY
A11	Ccl4	2.4648	(1.82, 3.11)	OKAY
A12	Ccl5	4.8967	(3.65, 6.14)	OKAY
B01	Cd14	8.1707	(6.87, 9.47)	OKAY
B02	Chuk	0.5275	(0.36, 0.69)	OKAY
B03	Crp	0.5409	(0.31, 0.77)	A
B04	Ctsg	0.5595	(0.03, 1.09)	B
B05	Cxcl1	32.9636	(15.84, 50.09)	OKAY
B06	Cxcl3	142.0708	(63.83, 220.31)	A
B07	Dmbt1	0.7767	(0.17, 1.39)	B
B08	Fadd	0.7557	(0.66, 0.85)	OKAY
B09	Hsp90aa1	0.8939	(0.75, 1.04)	OKAY
B10	Ifna9	0.7432	(0.53, 0.96)	C
B11	Ifnb1	0.5245	(0.18, 0.87)	B
B12	Ikbkb	1.001	(0.84, 1.16)	OKAY
C01	Il12a	1.1356	(0.30, 1.97)	B
C02	Il12b	2.4597	(1.53, 3.39)	OKAY
C03	Il18	0.8513	(0.77, 0.93)	OKAY
C04	Il1b	4.0665	(2.93, 5.20)	OKAY
C05	Il6	82.4892	(27.77, 137.21)	A
C06	Irak1	0.7067	(0.57, 0.84)	OKAY
C07	Irak3	5.6352	(0.00001, 18.35)	OKAY
C08	Irf5	0.8118	(0.68, 0.95)	OKAY
C09	Irf7	1.6561	(1.30, 2.01)	OKAY
C10	Jun	2.126	(1.42, 2.83)	OKAY
C11	Lbp	3.001	(2.09, 3.91)	OKAY
C12	Lcn2	146.4029	(31.30, 261.51)	OKAY
D01	Ltf	0.397	(0.28, 0.52)	OKAY

D02	Ly96	1.1317 (0.98, 1.28)	OKAY
D03	Lyz2	13.521 (11.29, 15.76)	OKAY
D04	Map2k1	1.028 (0.89, 1.16)	OKAY
D05	Map2k3	0.8482 (0.74, 0.96)	OKAY
D06	Map2k4	0.7274 (0.63, 0.82)	OKAY
D07	Map3k7	0.7773 (0.68, 0.88)	OKAY
D08	Mapk1	0.7432 (0.64, 0.84)	OKAY
D09	Mapk14	0.5836 (0.50, 0.66)	OKAY
D10	Mapk3	1.1084 (0.96, 1.26)	OKAY
D11	Mapk8	0.7162 (0.59, 0.84)	OKAY
D12	Mefv	9.8455 (5.67, 14.02)	A
E01	Mpo	5.865 (1.98, 9.75)	B
E02	Myd88	1.5481 (1.23, 1.87)	OKAY
E03	Naip1	7.8524 (3.19, 12.52)	OKAY
E04	Nfkb1	1.9951 (1.75, 2.24)	OKAY
E05	Nfkbia	1.7264 (1.05, 2.40)	OKAY
E06	Nlrc4	0.4587 (0.35, 0.57)	OKAY
E07	Nlrp1a	2.547 (1.95, 3.15)	OKAY
E08	Nlrp3	4.0087 (2.89, 5.12)	OKAY
E09	Nod1	0.9411 (0.75, 1.13)	OKAY
E10	Nod2	0.5779 (0.44, 0.71)	OKAY
E11	Pik3ca	0.6387 (0.55, 0.73)	OKAY
E12	Prtn3	1.5502 (0.91, 2.19)	A
F01	Pstpip1	1.4361 (0.00001, 2.93)	OKAY
F02	Pycard	2.0693 (1.84, 2.29)	OKAY
F03	Rac1	0.9239 (0.74, 1.11)	OKAY
F04	Rela	1.6262 (1.45, 1.80)	OKAY
F05	Ripk1	0.9173 (0.81, 1.02)	OKAY
F06	Ripk2	1.0525 (0.89, 1.22)	OKAY
F07	Slc11a1	3.959 (3.19, 4.73)	OKAY
F08	Slpi	2.4688 (0.40, 4.54)	OKAY
F09	Sugt1	1.0173 (0.89, 1.15)	OKAY
F10	Ticam1	0.8828 (0.73, 1.04)	OKAY
F11	Ticam2	4.9834 (4.04, 5.92)	OKAY
F12	Tirap	1.1549 (0.95, 1.36)	OKAY
G01	Tlr1	3.4987 (2.70, 4.29)	OKAY
G02	Tlr2	7.9345 (5.35, 10.52)	OKAY
G03	Tlr4	2.1742 (1.75, 2.60)	OKAY
G04	Tlr5	1.8346 (1.13, 2.54)	OKAY
G05	Tlr6	3.0933 (2.59, 3.60)	OKAY
G06	Tlr9	3.5011 (3.00, 4.00)	OKAY
G07	Tnf	5.3189 (3.38, 7.26)	OKAY
G08	Tnfrsf1a	1.7803 (1.60, 1.96)	OKAY
G09	Tollip	0.6751 (0.58, 0.77)	OKAY
G10	Traf6	0.7489 (0.66, 0.84)	OKAY
G11	Xiap	0.9422 (0.82, 1.07)	OKAY
G12	Zbp1	2.5511 (2.13, 2.98)	OKAY
H01	Actb	1.3111 (1.16, 1.46)	OKAY
H02	B2m	1.7998 (1.45, 2.15)	OKAY

H03	Gapdh	0.6581 (0.61, 0.71)	OKAY
H04	Gusb	0.8375 (0.74, 0.93)	OKAY
H05	Hsp90ab1	0.7689 (0.71, 0.83)	OKAY
H06	MGDC	0.7432 (0.53, 0.96)	C
H07	RTC	0.6992 (0.58, 0.82)	OKAY
H08	RTC	0.7018 (0.58, 0.83)	OKAY
H09	RTC	0.7106 (0.58, 0.85)	OKAY
H10	PPC	0.7262 (0.45, 1.01)	OKAY
H11	PPC	0.7494 (0.53, 0.97)	OKAY
H12	PPC	0.7683 (0.53, 1.01)	OKAY

		**2^(-Avg.(Delta(Ct))**	
Position	Symbol	Control Grc	Kidney stones
A01	Akt1	0.063519	0.079154
A02	Apcs	0.001856	0.000598
A03	Birc3	0.011665	0.036066
A04	Bpi	0.000017	0.000006
A05	Camp	0.000088	0.000086
A06	Card6	0.007977	0.007094
A07	Card9	0.001274	0.00188
A08	Casp1	0.002024	0.003493
A09	Casp8	0.06448	0.069966
A10	Ccl3	0.000449	0.001365
A11	Ccl4	0.000712	0.001754
A12	Ccl5	0.004927	0.024127
B01	Cd14	0.008539	0.069772
B02	Chuk	0.500578	0.264035
B03	Crp	0.000113	0.000061
B04	Ctsg	0.000009	0.000005
B05	Cxcl1	0.000416	0.013724
B06	Cxcl3	0.000006	0.000849
B07	Dmbt1	0.000004	0.000003
B08	Fadd	0.032464	0.024532
B09	Hsp90aa1	0.66281	0.592464
B10	Ifna9	0.000003	0.000002
B11	Ifnb1	0.00007	0.000037
B12	Ikbkb	0.04915	0.0492
C01	Il12a	0.00003	0.000034
C02	Il12b	0.000128	0.000315
C03	Il18	0.007264	0.006184
C04	Il1b	0.001367	0.005558
C05	Il6	0.000024	0.001971
C06	Irak1	0.109702	0.077525
C07	Irak3	0.000867	0.004886
C08	Irf5	0.025589	0.020772
C09	Irf7	0.016805	0.02783
C10	Jun	0.051713	0.109941

C11	Lbp	0.002807	0.008424
C12	Lcn2	0.004115	0.602403
D01	Ltf	0.000506	0.000201
D02	Ly96	0.012076	0.013667
D03	Lyz2	0.029598	0.400202
D04	Map2k1	0.065835	0.067677
D05	Map2k3	0.0758	0.064293
D06	Map2k4	0.055553	0.040408
D07	Map3k7	0.061568	0.047855
D08	Mapk1	0.227667	0.1692
D09	Mapk14	0.127774	0.074573
D10	Mapk3	0.063299	0.07016
D11	Mapk8	0.028524	0.02043
D12	Mefv	0.000044	0.000438
E01	Mpo	0.000003	0.00002
E02	Myd88	0.002998	0.004641
E03	Naip1	0.000169	0.001329
E04	Nfkb1	0.028723	0.057305
E05	Nfkbia	0.081052	0.139932
E06	Nlrc4	0.008393	0.003849
E07	Nlrp1a	0.00032	0.000815
E08	Nlrp3	0.000248	0.000993
E09	Nod1	0.009877	0.009296
E10	Nod2	0.002036	0.001177
E11	Pik3ca	0.092248	0.058916
E12	Prtn3	0.000093	0.000144
F01	Pstpip1	0.003488	0.005009
F02	Pycard	0.016535	0.034216
F03	Rac1	0.481297	0.444668
F04	Rela	0.019281	0.031354
F05	Ripk1	0.02024	0.018566
F06	Ripk2	0.006638	0.006987
F07	Slc11a1	0.001983	0.007849
F08	Slpi	0.000451	0.001113
F09	Sugt1	0.091505	0.093092
F10	Ticam1	0.01841	0.016252
F11	Ticam2	0.000528	0.002629
F12	Tirap	0.00566	0.006537
G01	Tlr1	0.001307	0.004571
G02	Tlr2	0.00165	0.013092
G03	Tlr4	0.004211	0.009155
G04	Tlr5	0.000356	0.000653
G05	Tlr6	0.000513	0.001587
G06	Tlr9	0.001829	0.006402
G07	Tnf	0.000253	0.001346
G08	Tnfrsf1a	0.085773	0.152703
G09	Tollip	0.080865	0.054591
G10	Traf6	0.050067	0.037494
G11	Xiap	0.036063	0.033979

G12	Zbp1	0.003111	0.007937
H01	Actb	2.313376	3.033115
H02	B2m	1.389918	2.501511
H03	Gapdh	3.313448	2.180713
H04	Gusb	0.048923	0.040973
H05	Hsp90ab1	1.918528	1.475087
H06	MGDC	0.000003	0.000002
H07	RTC	0.110721	0.077417
H08	RTC	0.113571	0.079704
H09	RTC	0.110465	0.078498
H10	PPC	0.627781	0.455903
H11	PPC	0.630689	0.472636
H12	PPC	0.639493	0.491342

		**AVG Delta(Ct) (Ct(GOI) - Ave Ct(HK(**		**Standard Deviation**	
Position	Symbol	Control Grc	Kidney stones	Control Group	Kidney stones
A01	Akt1	3.976667	3.6592	0.240432	0.089606
A02	Apcs	9.073333	10.7072	0.116342	0.854362
A03	Birc3	6.421667	4.7932	0.168587	0.033722
A04	Bpi	15.878333	17.3592	1.110051	1.047525
A05	Camp	13.476667	13.5012	0.926978	0.599711
A06	Card6	6.97	7.1392	0.206351	0.411751
A07	Card9	9.616667	9.0552	0.281964	0.277076
A08	Casp1	8.948333	8.1612	0.264903	0.250965
A09	Casp8	3.955	3.8372	0.164277	0.131587
A10	Ccl3	11.12	9.5172	0.543622	0.507469
A11	Ccl4	10.456667	9.1552	0.333406	0.304994
A12	Ccl5	7.665	5.3732	0.20768	0.373199
B01	Cd14	6.871667	3.8412	0.273081	0.080207
B02	Chuk	0.998333	1.9212	0.184102	0.493045
B03	Crp	13.106667	13.9932	0.548605	0.47861
B04	Ctsg	16.843333	17.6812	1.532569	0.700466
B05	Cxcl1	11.23	6.1872	0.8779	0.297717
B06	Cxcl3	17.351667	10.2012	0.856624	0.458381
B07	Dmbt1	17.916667	18.2812	0.90654	0.996807
B08	Fadd	4.945	5.3492	0.15193	0.154561
B09	Hsp90aa1	0.593333	0.7552	0.23725	0.157674
B10	Ifna9	18.215	18.6432	0.228549	0.421945
B11	Ifnb1	13.798333	14.7292	0.556127	0.966323
B12	Ikbkb	4.346667	4.3452	0.242964	0.136606
C01	Il12a	15.036667	14.8532	0.578307	1.085775
C02	Il12b	12.931667	11.6332	0.624197	0.248631
C03	Il18	7.105	7.3372	0.138805	0.09479
C04	Il1b	9.515	7.4912	0.29393	0.372295
C05	Il6	15.353333	8.9872	1.109432	0.407989
C06	Irak1	3.188333	3.6892	0.126386	0.297337
C07	Irak3	10.171667	7.6772	4.042835	0.403925

C08	Irf5	5.288333	5.5892	0.273535	0.104255
C09	Irf7	5.895	5.1672	0.327113	0.190723
C10	Jun	4.273333	3.1852	0.540681	0.226188
C11	Lbp	8.476667	6.8912	0.462627	0.264657
C12	Lcn2	7.925	0.7312	0.321314	1.26039
D01	Ltf	10.948333	12.2812	0.366657	0.365203
D02	Ly96	6.371667	6.1932	0.147368	0.171164
D03	Lyz2	5.078333	1.3212	0.193736	0.206793
D04	Map2k1	3.925	3.8852	0.225075	0.070719
D05	Map2k3	3.721667	3.9592	0.196177	0.12708
D06	Map2k4	4.17	4.6292	0.120486	0.188942
D07	Map3k7	4.021667	4.3852	0.160092	0.150536
D08	Mapk1	2.135	2.5632	0.18709	0.14487
D09	Mapk14	2.968333	3.7452	0.174601	0.157293
D10	Mapk3	3.981667	3.8332	0.213985	0.099485
D11	Mapk8	5.131667	5.6132	0.158586	0.25412
D12	Mefv	14.456667	11.1572	0.715418	0.247982
E01	Mpo	18.143333	15.5912	0.30976	1.054241
E02	Myd88	8.381667	7.7512	0.207107	0.282654
E03	Naip1	12.528333	9.5552	0.44806	0.887942
E04	Nfkb1	5.121667	4.1252	0.159642	0.134318
E05	Nfkbia	3.625	2.8372	0.691886	0.137496
E06	Nlrc4	6.896667	8.0212	0.185234	0.343064
E07	Nlrp1a	11.61	10.2612	0.302702	0.272733
E08	Nlrp3	11.978333	9.9752	0.361737	0.317587
E09	Nod1	6.661667	6.7492	0.263919	0.233301
E10	Nod2	8.94	9.7312	0.217653	0.336011
E11	Pik3ca	3.438333	4.0852	0.174532	0.179447
E12	Prtn3	13.391667	12.7592	0.474086	0.529839
F01	Pstpip1	8.163333	7.6412	1.870285	0.146742
F02	Pycard	5.918333	4.8692	0.092895	0.158016
F03	Rac1	1.055	1.1692	0.196354	0.27322
F04	Rela	5.696667	4.9952	0.172695	0.073764
F05	Ripk1	5.626667	5.7512	0.10442	0.163136
F06	Ripk2	7.235	7.1612	0.239505	0.143782
F07	Slc11a1	8.978333	6.9932	0.102672	0.306916
F08	Slpi	11.115	9.8112	1.372371	0.581088
F09	Sugt1	3.45	3.4252	0.178204	0.13282
F10	Ticam1	5.763333	5.9432	0.265886	0.158421
F11	Ticam2	10.888333	8.5712	0.225401	0.232235
F12	Tirap	7.465	7.2572	0.275512	0.149349
G01	Tlr1	9.58	7.7732	0.348971	0.194903
G02	Tlr2	9.243333	6.2552	0.178593	0.510334
G03	Tlr4	7.891667	6.7712	0.313186	0.149577
G04	Tlr5	11.456667	10.5812	0.604297	0.316106
G05	Tlr6	10.928333	9.2992	0.267861	0.112691
G06	Tlr9	9.095	7.2872	0.205511	0.140233
G07	Tnf	11.948333	9.5372	0.557628	0.319336
G08	Tnfrsf1a	3.543333	2.7112	0.143525	0.095672

G09	Tollip	3.628333	4.1952	0.21994	0.112655
G10	Traf6	4.32	4.7372	0.103174	0.17394
G11	Xiap	4.793333	4.8792	0.208594	0.107001
G12	Zbp1	8.328333	6.9772	0.265657	0.128939
H01	Actb	-1.21	-1.6008	0.113652	0.157858
H02	B2m	-0.475	-1.3228	0.228164	0.243157
H03	Gapdh	-1.728333	-1.1248	0.097803	0.089951
H04	Gusb	4.353333	4.6092	0.200278	0.045268
H05	Hsp90ab1	-0.94	-0.5608	0.068387	0.10756
H06	MGDC	18.215	18.6432	0.228549	0.421945
H07	RTC	3.175	3.6912	0.191507	0.226038
H08	RTC	3.138333	3.6492	0.191597	0.233172
H09	RTC	3.178333	3.6712	0.210289	0.246482
H10	PPC	0.671667	1.1332	0.240178	0.595951
H11	PPC	0.665	1.0812	0.226581	0.440889
H12	PPC	0.645	1.0252	0.246517	0.469267
