# Supplementary material for: The Interaction between Enterobacteriaceae and Calcium Oxalate Deposits
Source: PLoS One. 2015 Oct 8;10(10):e0139575. doi: 10.1371/journal.pone.0139575 (PMC4598009; doi:10.1371/journal.pone.0139575)
Supplement: S1 Table — (DOCX) [file pone.0139575.s004.docx]

| **Supplemental File S3: RT^2^ Profiler Antibacterial Response Array Gene Table** | | | | |
| --- | --- | --- | --- | --- |
| **PCR array catalog #** | | **PAMM-148Z** | | |
| **Position** | **Unigene** | **Refseq** | **Symbol** | **Description** |
| A01 | Mm.6645 | NM_009652 | Akt1 | Thymoma viral proto-oncogene 1 |
| A02 | Mm.330510 | NM_011318 | Apcs | Serum amyloid P-component |
| A03 | Mm.2026 | NM_007464 | Birc3 | Baculoviral IAP repeat-containing 3 |
| A04 | Mm.260883 | NM_177850 | Bpi | Bactericidal permeablility increasing protein |
| A05 | Mm.3834 | NM_009921 | Camp | Cathelicidin antimicrobial peptide |
| A06 | Mm.31252 | NM_001163138 | Card6 | Caspase recruitment domain family, member 6 |
| A07 | Mm.330064 | NM_001037747 | Card9 | Caspase recruitment domain family, member 9 |
| A08 | Mm.1051 | NM_009807 | Casp1 | Caspase 1 |
| A09 | Mm.336851 | NM_009812 | Casp8 | Caspase 8 |
| A10 | Mm.1282 | NM_011337 | Ccl3 | Chemokine (C-C motif) ligand 3 |
| A11 | Mm.244263 | NM_013652 | Ccl4 | Chemokine (C-C motif) ligand 4 |
| A12 | Mm.284248 | NM_013653 | Ccl5 | Chemokine (C-C motif) ligand 5 |
| B01 | Mm.3460 | NM_009841 | Cd14 | CD14 antigen |
| B02 | Mm.3996 | NM_007700 | Chuk | Conserved helix-loop-helix ubiquitous kinase |
| B03 | Mm.28767 | NM_007768 | Crp | C-reactive protein, pentraxin-related |
| B04 | Mm.4858 | NM_007800 | Ctsg | Cathepsin G |
| B05 | Mm.21013 | NM_008176 | Cxcl1 | Chemokine (C-X-C motif) ligand 1 |
| B06 | Mm.244289 | NM_203320 | Cxcl3 | Chemokine (C-X-C motif) ligand 3 |
| B07 | Mm.4138 | NM_007769 | Dmbt1 | Deleted in malignant brain tumors 1 |
| B08 | Mm.5126 | NM_010175 | Fadd | Fas (TNFRSF6)-associated via death domain |
| B09 | Mm.315997 | NM_010480 | Hsp90aa1 | Heat shock protein 90, alpha (cytosolic), class A member 1 |
| B10 | Mm.377092 | NM_010507 | Ifna9 | Interferon alpha 9 |
| B11 | Mm.1245 | NM_010510 | Ifnb1 | Interferon beta 1, fibroblast |
| B12 | Mm.277886 | NM_010546 | Ikbkb | Inhibitor of kappaB kinase beta |
| C01 | Mm.103783 | NM_008351 | Il12a | Interleukin 12A |
| C02 | Mm.239707 | NM_008352 | Il12b | Interleukin 12B |
| C03 | Mm.1410 | NM_008360 | Il18 | Interleukin 18 |
| C04 | Mm.222830 | NM_008361 | Il1b | Interleukin 1 beta |
| C05 | Mm.1019 | NM_031168 | Il6 | Interleukin 6 |
| C06 | Mm.38241 | NM_008363 | Irak1 | Interleukin-1 receptor-associated kinase 1 |
| C07 | Mm.146194 | NM_028679 | Irak3 | Interleukin-1 receptor-associated kinase 3 |
| C08 | Mm.6479 | NM_012057 | Irf5 | Interferon regulatory factor 5 |
| C09 | Mm.3233 | NM_016850 | Irf7 | Interferon regulatory factor 7 |
| C10 | Mm.275071 | NM_010591 | Jun | Jun oncogene |
| C11 | Mm.218846 | NM_008489 | Lbp | Lipopolysaccharide binding protein |
| C12 | Mm.9537 | NM_008491 | Lcn2 | Lipocalin 2 |
| D01 | Mm.282359 | NM_008522 | Ltf | Lactotransferrin |
| D02 | Mm.116844 | NM_016923 | Ly96 | Lymphocyte antigen 96 |
| D03 | Mm.45436 | NM_017372 | Lyz2 | Lysozyme 2 |
| D04 | Mm.248907 | NM_008927 | Map2k1 | Mitogen-activated protein kinase kinase 1 |
| D05 | Mm.18494 | NM_008928 | Map2k3 | Mitogen-activated protein kinase kinase 3 |
| D06 | Mm.412922 | NM_009157 | Map2k4 | Mitogen-activated protein kinase kinase 4 |
| D07 | Mm.258589 | NM_172688 | Map3k7 | Mitogen-activated protein kinase kinase kinase 7 |
| D08 | Mm.196581 | NM_011949 | Mapk1 | Mitogen-activated protein kinase 1 |
| D09 | Mm.311337 | NM_011951 | Mapk14 | Mitogen-activated protein kinase 14 |
| D10 | Mm.8385 | NM_011952 | Mapk3 | Mitogen-activated protein kinase 3 |
| D11 | Mm.21495 | NM_016700 | Mapk8 | Mitogen-activated protein kinase 8 |
| D12 | Mm.143718 | NM_019453 | Mefv | Mediterranean fever |
| E01 | Mm.4668 | NM_010824 | Mpo | Myeloperoxidase |
| E02 | Mm.213003 | NM_010851 | Myd88 | Myeloid differentiation primary response gene 88 |
| E03 | Mm.6898 | NM_008670 | Naip1 | NLR family, apoptosis inhibitory protein 1 |
| E04 | Mm.256765 | NM_008689 | Nfkb1 | Nuclear factor of kappa light polypeptide gene enhancer in B-cells 1, p105 |
| E05 | Mm.170515 | NM_010907 | Nfkbia | Nuclear factor of kappa light polypeptide gene enhancer in B-cells inhibitor, alpha |
| E06 | Mm.311884 | NM_001033367 | Nlrc4 | NLR family, CARD domain containing 4 |
| E07 | Mm.240227 | NM_001004142 | Nlrp1a | NLR family, pyrin domain containing 1A |
| E08 | Mm.54174 | NM_145827 | Nlrp3 | NLR family, pyrin domain containing 3 |
| E09 | Mm.28498 | NM_172729 | Nod1 | Nucleotide-binding oligomerization domain containing 1 |
| E10 | Mm.222633 | NM_145857 | Nod2 | Nucleotide-binding oligomerization domain containing 2 |
| E11 | Mm.260521 | NM_008839 | Pik3ca | Phosphatidylinositol 3-kinase, catalytic, alpha polypeptide |
| E12 | Mm.2364 | NM_011178 | Prtn3 | Proteinase 3 |
| F01 | Mm.2534 | NM_011193 | Pstpip1 | Proline-serine-threonine phosphatase-interacting protein 1 |
| F02 | Mm.24163 | NM_023258 | Pycard | PYD and CARD domain containing |
| F03 | Mm.469963 | NM_009007 | Rac1 | RAS-related C3 botulinum substrate 1 |
| F04 | Mm.249966 | NM_009045 | Rela | V-rel reticuloendotheliosis viral oncogene homolog A (avian) |
| F05 | Mm.374799 | NM_009068 | Ripk1 | Receptor (TNFRSF)-interacting serine-threonine kinase 1 |
| F06 | Mm.112765 | NM_138952 | Ripk2 | Receptor (TNFRSF)-interacting serine-threonine kinase 2 |
| F07 | Mm.2913 | NM_013612 | Slc11a1 | Solute carrier family 11 (proton-coupled divalent metal ion transporters), member 1 |
| F08 | Mm.371583 | NM_011414 | Slpi | Secretory leukocyte peptidase inhibitor |
| F09 | Mm.18972 | NM_026474 | Sugt1 | SGT1, suppressor of G2 allele of SKP1 (S. cerevisiae) |
| F10 | Mm.203952 | NM_174989 | Ticam1 | Toll-like receptor adaptor molecule 1 |
| F11 | Mm.149280 | NM_173394 | Ticam2 | Toll-like receptor adaptor molecule 2 |
| F12 | Mm.23987 | NM_054096 | Tirap | Toll-interleukin 1 receptor (TIR) domain-containing adaptor protein |
| G01 | Mm.273024 | NM_030682 | Tlr1 | Toll-like receptor 1 |
| G02 | Mm.87596 | NM_011905 | Tlr2 | Toll-like receptor 2 |
| G03 | Mm.38049 | NM_021297 | Tlr4 | Toll-like receptor 4 |
| G04 | Mm.116894 | NM_016928 | Tlr5 | Toll-like receptor 5 |
| G05 | Mm.42146 | NM_011604 | Tlr6 | Toll-like receptor 6 |
| G06 | Mm.44889 | NM_031178 | Tlr9 | Toll-like receptor 9 |
| G07 | Mm.1293 | NM_013693 | Tnf | Tumor necrosis factor |
| G08 | Mm.474976 | NM_011609 | Tnfrsf1a | Tumor necrosis factor receptor superfamily, member 1a |
| G09 | Mm.103551 | NM_023764 | Tollip | Toll interacting protein |
| G10 | Mm.292729 | NM_009424 | Traf6 | Tnf receptor-associated factor 6 |
| G11 | Mm.259879 | NM_009688 | Xiap | X-linked inhibitor of apoptosis |
| G12 | Mm.116687 | NM_021394 | Zbp1 | Z-DNA binding protein 1 |
| H01 | Mm.391967 | NM_007393 | Actb | Actin, beta |
| H02 | Mm.163 | NM_009735 | B2m | Beta-2 microglobulin |
| H03 | Mm.304088 | NM_008084 | Gapdh | Glyceraldehyde-3-phosphate dehydrogenase |
| H04 | Mm.3317 | NM_010368 | Gusb | Glucuronidase, beta |
| H05 | Mm.2180 | NM_008302 | Hsp90ab1 | Heat shock protein 90 alpha (cytosolic), class B member 1 |
